# Surgical site infection due to *Mycobacterium wolinskyi* after inguinal hernia surgery: A case report and review of the literature

**DOI:** 10.1016/j.jctube.2026.100596

**Published:** 2026-03-13

**Authors:** Yoshitaka Wakabayashi, Shin Nakayama, Ai Yamamoto, Shunya Suzuki, Junpei Sasaki, Miwa Asahara, Takeyuki Misawa, Takatoshi Kitazawa

**Affiliations:** a Department of Internal Medicine, Teikyo University School of Medicine, Tokyo, Japan; b Department of Laboratory Medicine, Teikyo University Hospital, Itabashi, Tokyo, Japan; c Department of Surgery, Teikyo University School of Medicine, 2-11-1 Kaga, Itabashi-Ku, Tokyo 173-8605, Japan

**Keywords:** Mycobacterium wolinskyi, Rapidly growing mycobacteria, Non-tuberculous mycobacteria, NTM, Surgical site infection

## Abstract

*Mycobacterium wolinskyi* (MW) is a rapidly growing mycobacterium that was first identified in 1999 using 16S rRNA gene sequencing. Although the number of reported cases of MW infection has been increasing in recent years, surgical site infections (SSIs) caused by MW remain rare, and standardized treatment regimens and durations have not yet been established. Herein, we report a case of SSI caused by MW after transabdominal preperitoneal repair intervention, along with a review of SSI cases caused by MW. An 86-year-old woman underwent transabdominal preperitoneal repair of an inguinal hernia with a synthetic mesh at another hospital. As the patient developed an SSI, drainage was performed; however, no pathogens were identified. At the the request of the patient, she was transferred to our hospital, and the infected mesh was removed. MW was isolated from the infected mesh. After completion of 24 weeks of oral minocycline and levofloxacin treatment, the patient was followed up for 1 year, and no recurrence was observed. We reviewed published cases of MW-associated SSI. All patients recieved a combination of antimicrobial therapies that involved two or more agents. Among the reported cases, fluoroquinolones were most frequently used (19 cases), followed by tetracyclines (15 cases). Our findings highlight that MW can be a potential causative organism of SSIs, particularly when routine cultures do not yield the typical pathogens. Prompt surgical intervention, including effective drainage, is crucial and can allow for a shorter duration of antimicrobial treatment.

## Highlights

• Surgical site infections caused by *Mycobacterium wolinskyi* are increasingly reported.
• This case involved a rare postoperative mesh infection after laparoscopic hernia repair.
• Complete mesh explantation was essential to achieve surgical source control.
• Adequate drainage and debridement may allow for shorter treatment duration.
• Fluoroquinolones, tetracyclines, and amikacin are commonly used in reported cases.

## 1 Introduction

Mycobacterium is a genus of bacteria in the family Mycobacteriaceae, within the phylum Actinomycetota [1]. It includes over 190 species, and *Mycobacterium tuberculosis* attracts most global concern owing to its high infectiousness.

*Mycobacterium wolinskyi* (MW) is a rapidly growing non-tuberculous mycobacterium (NTM) that was first identified in 1999 using 16S rRNA gene sequencing [1]. MW is generally found in natural water and soils, although one study reported the presence of MW in an operating room specifically associated with a cold air blaster and a microbially contaminated, self-contained water system [1]. Infections caused by MW are rare; however, the number of reported cases has increased in recent years. Therefore, sharing clinical experience and accumulating evidence are essential for developing effective treatment recommendations.

We report a case of MW-related mesh infection following hernia surgery before providing a review of previously published MW-associated SSI.

## 2 Case presentation

**Fig. 1:**
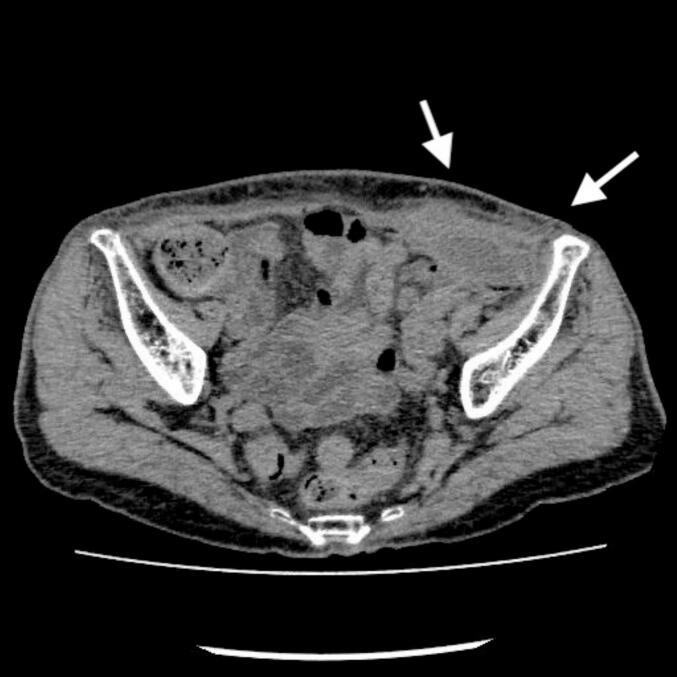
Abdominal/pelvic computed tomography image obtained on postoperative day 62 following mesh implantation for inguinal hernia repair. A massive abscess measuring 15.2 cm × 9.7 cm is observed in the Douglas pouch.

An 86-year-old woman underwent a transabdominal preperitoneal (TAPP) repair with a synthetic mesh at another hospital (defined as postoperative day [POD] 0). On POD 9, she developed a fever exceeding 38°C and persistent pain at the surgical site. Oral amoxicillin-clavulanate treatment (500 mg/125 mg three times daily) was initiated; however, no clinical improvement was observed. On POD 62, a computed tomography (CT) scan was performed due to persistent fever, worsening localized pain, and concern for a deep abscess. The CT scan revealed a massive abscess measuring 15.2 cm × 9.7 cm in the Douglas cavity (Fig. 1); therefore, the patient was readmitted to the hospital where the TAPP had been performed. Despite adequate abscess drainage and intravenous antibiotic therapy with piperacillin–tazobactam 4 g/0.5 g twice daily from POD 63 to POD 75 and meropenem 1 g twice daily from POD 76 to POD 83, antimicrobial therapy alone did not result in clinical improvement. Although Gram staining of the drainage fluid revealed the presence of leukocytes, no microorganisms were detected. At the patient’s request, she was transferred to our hospital on POD 102.

Physical examination on admission revealed the following findings: clear consciousness, blood pressure 148/85 mmHg, pulse rate 81 beats/min, body temperature 36.8 ℃, SpO2 98% (ambient air), and respiratory rate 16 breaths/min. Her abdomen was flat and soft, with a drain placed in the left inguinal region revealing serous drainage fluid; muscle guarding was absent. The laboratory findings on admission were as follows: white blood cell count 6,200/μL, platelet count 25.1 × 10^4^/μL, C-reactive protein 0.71 mg/dL; other parameters were unremarkable. The patient underwent mesh removal surgery on POD 104. The infected mesh was extracted through an open anterior approach. The hernia defect was closed primarily using the posterior rectus sheath and transversalis fascia, without placing a new mesh. Intraoperative cultures of the surgical site and removed mesh were initially performed as routine bacterial cultures, yielding growth on HK semisolid medium (Kyokuto Pharmaceutical Industrial, Japan) after seven days of incubation. Subculture on blood agar was subsequently performed, and visible colony formation was observed after an additional nine days. Microscopic examination of the colonies revealed acid-fast bacilli, raising suspicion of *Mycobacterium* species. *Mycobacterium wolinskyi* was identified using MALDI-TOF mass spectrometry (MALDI Biotyper Sirius, Bruker Daltonics, USA) with a score of 1.78. Subsequent genetic identification by 16S rRNA sequencing confirmed that the isolate was MW. Antimicrobial susceptibility testing was performed using BrothMIC RGM® (Kyokuto Pharmaceutical Industrial, Japan), the broth microdilution method according to the Clinical and Laboratory Standards Institute (CLSI) M24-A2 guidelines for rapidly growing mycobacteria. The results are shown in Table 2. Postoperative observation was initially performed; however, because a small amount of purulent drainage persisted at the surgical site, mesh explantation alone was considered insufficient for cure. Therefore, antimicrobial therapy targeting MW was initiated. Trimethoprim-sulfamethoxazole (TMP-SMX; 160 mg/400 mg twice daily), doxycycline (DOXY; 100 mg twice daily), and levofloxacin (LVFX; 500 mg once daily) were administered on POD 148 based on the susceptibility test. However, the patient developed a drug-induced rash within two weeks, prompting the discontinuation of all antibiotics.

As her rash improved, a new regimen consisting of LVFX 250 mg once daily, minocycline (MINO) 100 mg twice daily, and linezolid (LZD) 600 mg twice daily was initiated on POD 169. Although her platelet count was 20.8 × 10^4^/μL on POD169, and follow-up laboratory testing on POD 211 showed a decrease to 10.1 × 10^4^/μL. The three antibiotics were discontinued on the same day. She was subsequently referred to the Department of Internal Medicine on POD 233.

The general condition of the patient and laboratory data, including platelet count, improved at that time. Given the suspected causative agents for adverse reactions (TMP-SMX or DOXY for rash and LZD for thrombocytopenia), treatment was resumed with MINO 100 mg twice daily and LVFX 500 mg once daily on POD 254. The patient tolerated the new regimen well, without any significant side effects, and oral therapy was completed on POD 376. Follow-up visits did not show local recurrence, and CT one year after completion of treatment did not reveal any evidence of reinfection. Outpatient follow-up was concluded at that time.

## 3 Discussion

SSI rates vary widely worldwide, with pooled estimates approximately 11.8% in low- and middle-income countries [2]. In contrast, the overall SSI rate in the United States has been reported to be approximately 1.9% [3]. SSIs are often caused by gram-positive cocci such as *Staphylococcus aureus* and *Staphylococcus epidermidis* [4]. Recently, SSIs caused by mycobacteria have been increasingly reported as emerging pathogens [5]. The most commonly reported species of mycobacteria causing SSIs appears to be rapidly growing mycobacteria (RGM) such as *Mycobacterium fortuitum* and *Mycobacterium abscessus* [5]. Although MW is classified as an RGM, reports of SSI caused by this organism are rare.

When SSI is suspected, wound tissue samples are typically cultured on various standard microbiological media, including blood agar, MacConkey agar, and chocolate agar, to identify the causative pathogens [6]. Several mycobacterial species can grow on blood or chocolate agar; however, *Mycobacterium* spp. takes longer to culture [5]. In addition, other microorganisms such as streptococci can interfere with the culture of mycobacteria in patient samples [7]. Thus, specific media such as the liquid media Mycobacteria Growth Indicator Tube or the egg-based Ogawa medium are used for culturing *Mycobacterium* spp. [8]. Moreover, as these media are not routinely used, mycobacterial infections are often diagnosed late or are missed. Additionally, even after observing mycobacteria growth in specialized culture media, identifying specific species and obtaining susceptibility test results requires considerable time [9]. These factors render mycobacterial infections difficult to diagnose and treat. In the present case, MW was unexpectedly isolated from routine media, highlighting the importance of maintaining a high index of suspicion and close examination of mycobacteria in cases of persistent SSI, particularly when the response to conventional antibiotic therapy is poor.

Currently, no guidelines are available for the treatment of MW. Moreover, the optimal antibiotics and treatment durations for MW remain unknown. Although MW is classified as an RGM, it may exhibit different clinical and antimicrobial susceptibility characteristics compared with those of more commonly encountered RGM species. We reviewed the literature of MW-associated SSIs using PubMed, MEDLINE, and Google Scholar with the keywords (“*Mycobacterium wolinskyi*”) and identified 15 relevant reports [10], [11], [12], [13], [14], [15], [16], [17], [18], [19], [20], [21], [22], [23], [24]. We excluded one article from a cluster of five SSIs without specific information regarding patient characteristics. Table 1 summarizes the details of the remaining 19 patients for whom individual clinical information was available.

**Table 1:** Summary of clinical characteristics of 19 patients with *Mycobacterium wolinskyi*-associated surgical site infections.

Ref	Year	Age	Sex	Surgical details	Onset days after operation	Duration of treatment	Initial therapy	Subsequent therapy	Outcome
[10]	2024	49	F	LASIK surgery	2 months	6 months	DOXY, MFLX		Cured
[11]	2024	26	F	Bilateral breast augmentation	5 months	7 months	TMP-SMX, CPFX		Cured
[12]	2021	30	F	Liposuction, abdominoplasty, herniorrhaphy	1 month	N.D.	N.D.		N.D.
[13]	2021	63	F	CRT device implantation	1 month	6 weeks	DOXY, MFLX		Cured
[14]	2021	82	M	Aortic and mitral valve replacement	40 days	12 months	AMK, IPM/CS, CAM	CPFX, MINO	Cured
[15]	2019	62	M	Total knee arthroplasty	11 months	6 months	AMK, LZD, MFLX	LZD, MFLX	Cured
[16]	2017	66	M	Peritoneal dialysis catheter insertion	2 months	39 days	LVFX, MINO		Cured
[17]	2016	48	M	Aortic valve replacement	15 days	At least 6 months	AMK, LZD, MFLX, DOXY		Cured
[19]	2013	65	F	Total knee arthroplasty	3 weeks	More than 4 months	AMK, CPFX, DOXY	CPFX, DOXY	Cured
[18]	2013	29	F	Bilateral reductive mammoplasty	1 year	12 months	TMP-SMX, DOXY	CPFX, DOXY, AMK then CPFX, DOXY	Cured
[20]	2013	67	F	Peritoneal dialysis catheter insertion	2 months	4 weeks	MFLX, EM, TOB	MFLX, DOXY, LZD	Cured
[24]	2013	56	F	Facial cosmetic procedures	N.D.	5 months	CAM, AMK, CFPX	DOXY, CPFX	Cured
[23]	2012	65	F	Total knee replacement	N.D.	N.D.	AMK, CPFX, DOXY		Cured
[21]	2011	84	M	Aortic valve replacement, sternotomy	1 month	6 months	IPM/CS, TMP-SMX, MFLX	TMP-SMX, MFLX	Cured
[21]	2011	28	F	Lung transplantation	9 months	6 months	LZD, MFLX, CAM	MFLX, DOXY	Cured
[21]	2011	73	M	AICD placement	2 months	6 months	CPFX, MINO		Cured
[21]	2011	16	M	Valve-sparing aortic root replacement	8 months	On suppressive therapy	AMK, MFLX, DOXY	MFLX, DOXY	Stable
[21]	2011	78	M	CABG	2 months	On treatment	CAM, CFX, MFLX	TGC, TMP-SMX, MFLX	Stable
[22]	2006	83	F	Total hip replacement	4 months	6 months	MFLX, MINO, AMK	MFLX, MINO	Cured
This case	2025	86	F	Transabdominal preperitoneal repair	1 week	4 months	CAM, LVFX		Cured

Abbreviations: M, male; F, female; N.D., not described; AMK, amikacin; CAM, clarithromycin; CFX, cefoxitin; CPFX, ciprofloxacin; DOXY, doxycycline; EM, erythromycin; IPM/CS, imipenem/cilastatin; LVFX, levofloxacin; LZD, linezolid; MFLX, moxifloxacin; MINO, minocycline; TGC, tigecycline; TMP-SMX, trimethoprim-sulfamethoxazole; TOB, tobramycin; LASIK, laser-assisted in situ keratomileusis; CABG, coronary artery bypass graft; CRT, cardiac resynchronization therapy; AICD, automatic implantable cardioverter defibrillator.

**Table 2:** Antimicrobial susceptibility results of *Mycobacterium wolinskyi* isolates from reviewed cases.

Ref	Method of susceptibility tests	CFX	IPM	CPFX	MFLX	TMP-SMX	AMK	CAM	DOXY	LZD	TOB	MEPM
[10]	N.D.	N.D.	N.D.	N.D.	N.D.	N.D.	N.D.	N.D.	N.D.	N.D.	N.D.	N.D.
[11]	N.D.	I	I	S	S	S	S	R	S	S	N.D.	N.D.
[12]	N.D.	N.D.	N.D.	N.D.	N.D.	N.D.	N.D.	N.D.	N.D.	N.D.	N.D.	N.D.
[13]	N.D.	N.D.	R	N.D.	N.D.	N.D.	S	N.D.	S	N.D.	N.D.	N.D.
[14]	Broth microdilution	N.D.	S(4)	S(<0.5)	N.D.	S(<2/38)	S(<8)	R(>8)	N.D.	S(<4)	N.D.	N.D.
[15]	Broth microdilution	N.D.	N.D.	S(1)	S(<0.25)	N.D.	S(8)	N.D.	N.D.	S(2)	N.D.	N.D.
[16]	Broth microdilution	N.D.	I(8)	S(0.5)	S(<0.5)	S(<2/38)	S(4)	R(>32)	N.D.	S(3)	R(>16)	N.D.
[17]	N.D.	N.D.	N.D.	N.D.	N.D.	N.D.	N.D.	N.D.	N.D.	N.D.	N.D.	N.D.
[19]	N.D.	N.D.	R	S	N.D.	R	S	R	S	N.D.	N.D.	N.D.
[18]	Broth microdilution	I(64)	N.D.	S(1)	I(<2)	*R(>64)	N.D.	R(16)	I(4)	N.D.	R(32)	N.D.
[20]	N.D.	N.D.	N.D.	S	N.D.	S	S	N.D.	S	S	N.D.	N.D.
[24]	N.D.	I(64)	I(16)	S(<1)	S(<0.25)	R(16/304)	S(8)	IR(4–16)	S(2)	S(8)	N.D.	N.D.
[23]	Broth microdilution	R(>256)	R(>64)	S(1)	S(<0.125)	*R(>128)	S(16)	R(64)	S(0.5)	N.D.	R(>32)	N.D.
[21]	N.D.	I(64)	R(16)	S(1)	N.D.	S(0.25/4.75)	N.D.	R(>16)	0.5(S)	N.D.	R(>16)	N.D.
[21]	N.D.	I(64)	R(16)	S(1)	N.D.	S(16)	S(4)	R(>32)	0.5(S)	N.D.	R(32)	N.D.
[21]	N.D.	I(32)	R(16)	S(1)	N.D.	S(0.25/4.75)	S(4)	R(>32)	0.5(S)	N.D.	R(32)	N.D.
[21]	N.D.	I(64)	R(16)	S(1)	N.D.	S(0.25/4.75)	S(4)	R(>16)	0.5(S)	N.D.	R(>16)	N.D.
[21]	N.D.	I(32)	R(16)	S(1)	N.D.	S(0.5/9.5)	S(4)	R(>16)	0.5(S)	N.D.	R(>16)	N.D.
[22]	Disk diffusion and Etest	I(24)	S(3)	S(0.25)	S(0.12)	N.D.	S(2)	R(16)	N.D.	S(0.7)	R(24)	N.D.
This case	Broth microdilution	N.D.	R(32)	N.D.	S(<0.25)	*R(>152)	S(8)	R(>64)	S(1)	S(<1)	R(>16)	I(16)

Numbers in parentheses represent MICs. * MIC value represents sulfamethoxazole alone. Abbreviations: M, male; F, female; R, resistant; I, intermediate; S, susceptible; IR, inducible resistance; N.D., not described; AMK, amikacin; CAM, clarithromycin; CFX, cefoxitin; CPFX, ciprofloxacin; DOXY, doxycycline; EM, erythromycin; IPM, imipenem; MEPM, meropenem; LVFX, levofloxacin; LZD, linezolid; MFLX, moxifloxacin; MINO, minocycline; TGC, tigecycline; TMP-SMX, trimethoprim-sulfamethoxazole; TOB, tobramycin.

The reviewed sample consisted of 19 individuals (11 females, 8 males) with a median age of 63 years (interquartile range [IQR], 30–73 years). The median time from surgery to the appearance of the symptoms was 60 days (IQR, 30–195). However, the wide range observed across cases (7–365 days) highlights the variability in clinical presentation and suggests that timing alone is not sufficient to distinguish MW infections from other pathogens. The most frequent surgical category was cardiovascular surgery, accounting for seven cases. This was followed by cosmetic surgery (four cases) and orthopedic surgery (four cases). These findings suggest that MW infections occur in various surgical fields, including implant-related and clean surgical procedures. The 19 reviewed cases derived from various geographic regions, including the United States (n = 6), South Korea (n = 3), Japan (n = 2), France (n = 2), and one case each from Brazil, India, Iraq, Spain, Switzerland, and the Netherlands. This distribution suggests that MW infections are not restricted to a particular region and can occur worldwide.

The optimal duration of antimicrobial therapy for skin and soft tissue infections or SSIs caused by RGM remains undefined. A study on *M. fortuitum*, another RGM species, reported that cellulitis was successfully treated with a mean duration of 10.6 weeks of antimicrobial therapy, commonly involving trimethoprim-sulfadiazine, amikacin, and cefoxitin with generally favorable outcomes [25]. Among the 19 patients whose cases were reviewed and treatment duration was clearly described, 12 underwent antimicrobial therapy for at least 6 months. In contrast, three patients were treated for less than 3 months, and none of them relapsed. In one patient, long-term antimicrobial therapy was required because the indwelling prosthetic material could not be removed despite drainage [21].

Among the patients who underwent adequate drainage, even those with shorter treatment durations did not experience relapse, suggesting that surgical drainage plays a critical role in treatment success. Surgical interventions such as debridement were performed in all but one case, underscoring the crucial role of surgical management combined with antimicrobial therapy. In our case, infection was limited to the area where the surgical mesh was inserted; moreover, complete mesh removal with wound debridement markedly improved the local condition. This case highlights the importance of prompt drainage, surgical debridement, and removal of the infection source in the management of RGM infections.

In general, at least two antibiotics are used to treat localized infections, including skin infections caused by RGM [25]. Among the reported cases, fluoroquinolones were most frequently used (19 cases), followed by tetracyclines (15 cases). In this review, we summarized the antimicrobial susceptibility results of each case report based on the agents defined by the CLSI broth microdilution M24-A2 for RGM or disk diffusion and Etest (Table 2). Among antibiotics for which sensitivity was reported, most strains showed good sensitivity to fluoroquinolones, doxycycline, linezolid, and amikacin. Nine strains were sensitive to trimethoprim-sulfadiazine, whereas five were resistant. In addition, all strains were resistant to clarithromycin. This may be related to intrinsic or inducible macrolide resistance, possibly mediated by erm-family ribosomal methylase genes, as reported in *M. wolinskyi* and related rapidly growing mycobacteria, although the exact genetic basis was not investigated in the present study [26]. Although CLSI-based susceptibility testing is essential for guiding therapy, the 2020 ATS/ERS/ESCMID/IDSA guidelines acknowledged that several antimycobacterial agents exhibited a poor correlation between in vitro resistance and clinical outcomes [27]. Therefore, the use of antibiotics presenting resistant *in vitro* may still be justified in certain clinical contexts, particularly in multidrug regimens for NTM infections, especially when options are limited owing to adverse drug reactions to other agents.

In conclusion, SSIs refractory to conventional antibiotics or showing negative results on routine cultures should alert clinicians to the possibility of Mycobacterial infection, including MW. In addition, this case suggests that with appropriate surgical debridement and control of the infection source, the treatment duration for MW-associated SSI may be shortened.

## 4 Ethical statement

This case report was conducted in accordance with the ethical standards of the institutional research committee and with the 1964 Helsinki declaration and its later amendments. Informed consent was obtained from the patient for publication of this case report. The patient’s written consent was obtained. According to the institutional policy of Teikyo university, single-patient case reports are considered part of clinical care and are therefore not human-subjects research; consequently, the Teikyo university’s Ethics Review Committee did not review this case report. This work conformed to the Ethical Guidelines for Medical and Biological Research Involving Human Subjects issued in Japan by the Ministry of Health, Labour and Welfare. No ethics committee approval number is applicable.

## 5 Informed consent and patient details

The patient’s written consent was obtained. According to the institutional policy of Teikyo University, single-patient case reports are considered part of clinical care and are therefore not human-subjects research; consequently, the Ethics Review Committee of Teikyo University did not review this case report. This work was performed in accordance with the Ethical Guidelines for Medical and Biological Research Involving Human Subjects issued in Japan by the Ministry of Health, Labour and Welfare.

## Funding sources

None.

## CRediT authorship contribution statement

**Yoshitaka Wakabayashi:** Writing – review & editing, Writing – original draft, Methodology, Investigation, Formal analysis, Data curation, Conceptualization. **Shin Nakayama:** Writing – review & editing. **Ai Yamamoto:** Writing – review & editing. **Shunya Suzuki:** Writing – review & editing. **Junpei Sasaki:** Writing – review & editing. **Miwa Asahara:** Writing – review & editing. **Takeyuki Misawa:** Writing – review & editing. **Takatoshi Kitazawa:** Writing – review & editing.

## Declaration of competing interest

The authors declare that they have no known competing financial interests or personal relationships that could have appeared to influence the work reported in this paper.
